# A randomized double-blind placebo-controlled trial to investigate the effectiveness and safety of a novel green-lipped mussel extract -BioLex® -for managing pain in moderate to severe osteoarthritis of the hip and knee

**DOI:** 10.1186/s12906-017-1907-9

**Published:** 2017-08-22

**Authors:** Simon Stebbings, Andrew Gray, Anthony G. Schneiders, Andrew Sansom

**Affiliations:** 1 Department of Medicine, Dunedin School of Medicine, University of Otago, Dunedin, New Zealand; 2 Department of Preventive and Social Medicine, University of Otago, Dunedin, New Zealand; 3 School of Human Health and Social Sciences, Central Queensland University, Branyan, Australia; 4 Seperex Nutritionals, The Centre for Innovation, Dunedin, New Zealand

**Keywords:** Green lipped mussel extract, Randomized controlled trial, Osteoarthritis

## Abstract

**Background:**

Extracts from *perna canaliculus,* the Green Lipped Mussel (GLM) are widely used as a complimentary therapy by patients with osteoarthritis (OA). The current study investigated the potential of a novel GLM formulation as a treatment for OA. A randomized double-blind placebo-controlled trial was undertaken to assess potential impacts on pain and quality of life following 12 weeks of treatment.

**Methods:**

Eighty patients with moderate to severe OA of the hip or knee were randomized to receive either 600 mg of BioLex®-GLM daily or placebo for 12 weeks. Entry criteria included a minimum 100 mm Visual Analogue Scale pain score (VAS) of 30 mm at baseline.

The primary outcome was patient reported pain, measured by the Western Ontario and McMasters OA Index (WOMAC) pain subscale and VAS pain scale. Secondary outcomes included: quality of life (OAQol), total WOMAC score, WOMAC −20 responder criteria, and change in medication use over the study period.

Participants were assessed at baseline, 12 weeks (end of therapy) and 15 weeks (3-weeks post-intervention).

**Results:**

At week 12, there were no significant differences in VAS or WOMAC pain subscale between active and placebo groups, nor significant improvement in the WOMAC-20 responder criteria or OAQol. Joint stiffness (measured by WOMAC-B stiffness) in the GLM group improved compared with placebo (*p* = 0.046). There was a significant difference in paracetamol use between the GLM treated group and the placebo group after week 12 (*p* = 0.001).

**Conclusions:**

BioLex® -GLM extract did not confer clinical benefit in moderate to severe OA over the intervention period, however, a significant difference in paracetamol use in the post-intervention period was observed between the BioLex® -GLM group and placebo group. Higher doses and/or longer treatment periods are worthy of future investigation.

**Trial registration:**

Australia and New Zealand Clinical Trials Registry: no. ACTRN12611000256976.

## Background

Pain is a common experience for people with Osteoarthritis (OA) and frequently contributes to disability [1]. Pharmacological management of OA is aimed at relieving pain through the use of simple analgesics, nonsteroidal anti-inflammatory drugs (NSAIDS) and opiates [2, 3], however these therapies are associated with significant adverse effects, particularly with regular, long-term use [3]. Furthermore, pain is poorly managed in patients with arthritis especially in the older age group, and such patients frequently use complementary and alternative medicine (CAM) in an attempt to alleviate symptoms and improve their quality of life [4].

Dietary supplements are the most popular form of CAM used by patients with OA and include; vitamin supplements, fish oil and evening primrose oil [5]. In recent years glucosamine and chondroitin sulphate have become one of the most widely used CAM for OA although meta-analyses have failed to show consistent benefit in terms of pain relief [6].

New Zealand Green Lipped Mussel (GLM, *Perna canaliculus*) lipid extracts have shown promise as a treatment for OA [5] with several clinical trials showing benefit across a range of outcomes. However, some of these studies had methodological deficiencies and others have been small pilot studies [7, 8].

The anti-inflammatory properties of GLM lipids in particular, have been studied extensively and there are credible biological mechanisms to suggest other potential benefits of GLM for treating OA [7, 8]**.** In the current study, a novel, bioactive GLM lipid extract enriched in N-acylethanloamine (NAE) and long-chain omega-3 fatty acids (BioLex®-GLM) was selected. Most of the anti-inflammatory activity of GLM is known to reside in the lipid fraction [8]. The manufacturing process for GLM products varies widely and preparations used in clinical trials vary widely in the composition of bioactive compounds [9].

The purpose of the study was to conduct a randomized, double-blind, placebo controlled trial using BioLex®-GLM in patients with moderate to severe OA of the hip and knee, with participants identifying their index joint. The primary outcome selected was patient-evaluated pain at the index joint.

## Methods

Ethical approval for the study was obtained from the Lower South Regional Ethics Committee, New Zealand. All participants were over 18 years of age and provided written informed consent in accordance with the Declaration of Helsinki. Enrollment was over a 9 month period between August 2011 and April 2012.

Participants were recruited from databases of patients with OA (attending Dunedin Hospital, New Zealand) and from advertisements placed in local newspapers (see Results for details).

The study sponsors -Seperex Nutritionals- had input into the study design, provided the study medication and placebo but were not involved in the selection of clinical assessments or statistical analysis, which was undertaken independently by research staff at the University of Otago.

### Trial design

A double-blind, randomized, placebo-controlled study of 12 weeks duration was conducted. Primary analyses were conducted according to modified intention to treat principles (randomized and using all available data). Measurements were taken at baseline (0 weeks), 6 weeks, 12 weeks and 15 weeks. The protocol conformed to the CONSORT guidelines for the reporting of randomized controlled trials (RCT).

### Eligibility criteria

Participants fulfilled the American College of Rheumatology classification criteria for OA of the major lower limb weight bearing joints [10] [11] with pain affecting the hip or knee on most days of the past month and radiological changes (osteophytes and joint space narrowing) indicative of OA. Participants identified an index joint for the study. This joint was x-rayed and graded by a single experienced radiologist according to the Kellgren-Lawrence scale (0–4) [12].

Required entry criteria were pain in the groin, hip or knee region for more than 3 months with pain rated ≥30 mm in the last week on 100 mm visual analogue scale (VAS). A minimum level of disability (Health Assessment Questionnaire (HAQ) Disability Index [13]) was also defined for inclusion at 0.5 out of 3.

### Exclusion criteria

The following were exclusion criteria for the study: Hip or knee surgery within past 6 months, current or recent (in the last 3 months) oral or intra-articular corticosteroid therapy, inflammatory arthritis, a history of hip or knee joint replacement or osteotomy at the index joint, other previous hip or knee pathology such as a recent fracture (<3 months) at the index joint, other co-existent muscular, joint or neurological condition causing pain or affecting lower limb function; (e.g. trauma), current or recent (< 6 weeks) use of omega-3 fatty acid supplements. Any medical or physical impairment apart from hip or knee OA precluding safe participation in exercise/function based assessments such as uncontrolled hypertension, fibromyalgia, chronic fatigue syndrome, seafood hypersensitivity or allergy, major depressive illness or psychosis, pregnancy, and inter-current acute infections.

### Green lipped mussel extract and placebo

Both Biolex®-GLM extract and the placebo (corn oil) were administered in 150 mg dark brown, opaque gel capsules of identical appearance and odour, contained in blister packs of 25 capsules. Each subject was instructed to take four capsules (total 600 mg) once per day with food. The dose selected was based upon that of previous studies using different GLM lipid extracts [7, 8].

### Randomization

Trial allocation was performed with allocation concealment (opaque envelopes), using eight strata (all combinations of sex: male, female; arthritis: moderate, severe; and age: under 65, 65+) with random block sizes of 2 and 4 (equally likely). Researchers screening potential participants were not aware of the block sizes used. The statistician was blinded as to treatment group allocation until all main analyses, including per-protocol and subgroup analyses, were completed.

### Concomitant medications

All participants were asked to discontinue use of any complimentary or alternative medicines a minimum of 3 weeks before entry into the study. This included glucosamine preparations, chondroitin and fish oil supplements. Participants were asked to avoid starting new medications during the trial, especially non-steroidal anti-inflammatory drugs (NSAID), and analgesics, including all opiate derivatives, but could continue with their normal NSAID/analgesic doses.

Participants kept a diary of their analgesic and NSAID use, recording dose and frequency on a daily basis during the study. Participants could reduce their usual analgesic and/or NSAID dose if they felt improvement in their pain levels during the trial period. Doses of NSAIDs were standardized as a single unit score using the Assessment in Spondyloarthritis International Society (ASAS) NSAID dose equivalent score [14].

Other analgesics including paracetamol, opioids (morphine and analogues, dihydrocodeine, codeine) and tramadol were recorded by the participants.

### Assessment of adherence

Participants completed a daily adherence diary for their study medication and were asked to keep all empty packs and unused medication. A pill count was performed by the blinded assessor at 6 and 12 weeks to gauge adherence.

### Outcome measures

Outcome measures were selected according to the recommendations of the Osteoarthritis Research Society International (OARSI) and OMERACT (Outcome measures in Rheumatology Clinical Trials) [15]. Pain at the index joint was selected as the primary outcome measure. This was assessed using two standard instruments: 1) The Western Ontario McMasters Osteoarthritis Index (WOMAC) [16] pain scale – with each of the 5 questions on a 5 point Likert scale (Subscale A- range 0–20). 2) A 100 mm visual analogue pain scale. Secondary outcomes were: Patient Global assessment on a 10 point numerical rating scale (NRS), Assessor’s Global assessment on a 10 point NRS scale, WOMAC subscales – stiffness (Subscale B scored 0–8) and disability (subscale C, scored 0–68), WOMAC total score (5 point Likert scale version) (range 0–96), Osteoarthritis Quality of life score (OAQoL) a 22 item validated quality of life measure with answers rated ‘yes’ or ‘no’ (range 0–22) [17], C- reactive protein (m/L) (Normal range < 1 mg/L), and Anglicized version of the HAQ –disability index (range 0–3). Participants kept a diary of their analgesic/ anti-inflammatory medication use over the study period, and changes in medication use was ascertained and evaluated as an outcome.

### Physical outcome measures

The following functional performance tests were employed: (i) The stair climb test involved timing how long it took participants to ascend and descend six steps at their own pace [18] (ii). The 30 s sit-to-stand test evaluated the number of times participants could repeatedly rise to a full standing position from sitting in 30s [19]. (iii) Walking performance was assessed by calculating walking velocity (m/s) as participants walk 20 m [20] (iv) Dynamic balance was assessed by using the timed Tandem Gait test over a six metre track [21].

### Recording of adverse events

All adverse events (AE) were recorded, together with details of seriousness and likelihood of attribution to the study agent, in accordance with National Institute for Health (NIH) Guidelines. For each adverse event, a grade of severity was attributed (1–3). Duration of AE was recorded and together with information regarding any interruption of the study medication (including the duration of any such interruption) and finally whether the AE resulted in the patient withdrawing from the study. Participants were questioned regarding AE’s at each visit, given contact details for the study co-ordinator and encouraged to contact the research team if they experienced any adverse events between visits. Any serious adverse events were immediately notified to the Ethics committee, sponsor and principal investigator.

### Timing of outcome measures

Measurements were performed in the treatment and control group at the following visits: (i) Screening (telephone) (ii) Pre-enrollment visit, (iii) Enrollment (Week 0), (iv) Compliance (Week 6), (v) Treatment completion (Week 12) and (vi) Study termination (Week 15).

### Responder criteria

Two definitions of patient response from baseline were used as described by Bellamy et al. [22]: (1) at least a 20% reduction in WOMAC pain score (WOMAC-A 20); (2) at least a 20% reduction in WOMAC total score (WOMAC-ABC 20). Responders at the 50 and 70% were also calculated to detect treatment group differences.

### Statistical methods

#### Power analysis

In order to provide 80% power to detect the minimum clinically meaningful difference in VAS pain scores measured (13 mm) at the two-sided 0.05 level, assuming a standard deviation of 23 mm, a correlation between baseline and follow-up scores of 0.5 or higher, and allowing for attrition/missing data of up to 10%, 42 participants were needed per group (84 overall). This sample size permitted the detection of an odds ratio for decreasing medication in the intervention group compared to the control group of 4.9, using McNemar’s test for paired binary data and assuming 50% of pairs are disconcordant (equivalent to a difference in proportions of 0.33, or 8.6% versus 41.4%). Criteria for minimal clinically important improvement (MCII) of pain, patient’s global assessment of disease activity, and functional impairment were preset according to OARSI response criteria [23].

Compliance was compared between the two groups using a Mann-Whitney-Wilcoxon test. For outcomes with interim measures available, linear mixed models with a random subject effect were used to compare groups, adjusting for baseline values where available. Linear contrasts were used to compare changes between time points and groups where the interaction between group and time was significant. For outcomes with only week 12 measures available, regression models adjusting for baseline values were used. In all cases, model residuals were checked for evidence of non-normality or heteroscedasticity and log-transformations investigated and used where this improved any unsatisfactory residuals. A per protocol secondary analysis was planned a priori using 80% compliance and the proportion achieving this was compared between groups using a Chi-squared test. Prior to statistical modeling, but after data collection and descriptives, subgroup analyses were planned for those with baseline lower levels of pain and joint damage. For comparing rates of improvement in WOMAC scores (WOMAC-20, WOMAC-50, and WOMAC-70), Chi-square tests were used with Fisher’s exact test employed where more than 20% of cells had expected counts below 5. Two sets of variables were considered as overall indicators: QoL (pain VAS, WOMAC-total, OA QoL, global patient assessment and global physician assessment) and functioning (stair climbing, sit-to-stand, dynamic balance, and walking). As STS goes in the opposite direction to the other two variables in that set, this score was reversed by subtracting observed values from the maximal achieved (16 repetitions). All variables were normalized using the relevant baseline mean and standard deviation with lower scores being preferable. The resulting normalized week 12 scores were then modeled using a random effect to accommodate the repeated measures and adjusting for baseline values. Statistical significance was determined by two-sided *p* < 0.05 in all cases. All analyses were performed using Stata 12.1. The post-hoc power of the study is communicated through 95% confidence intervals, showing lower and upper limits of effect sizes consistent with the observed data, which have been provided as appropriate in the results.

## Results

**Fig. 1:**
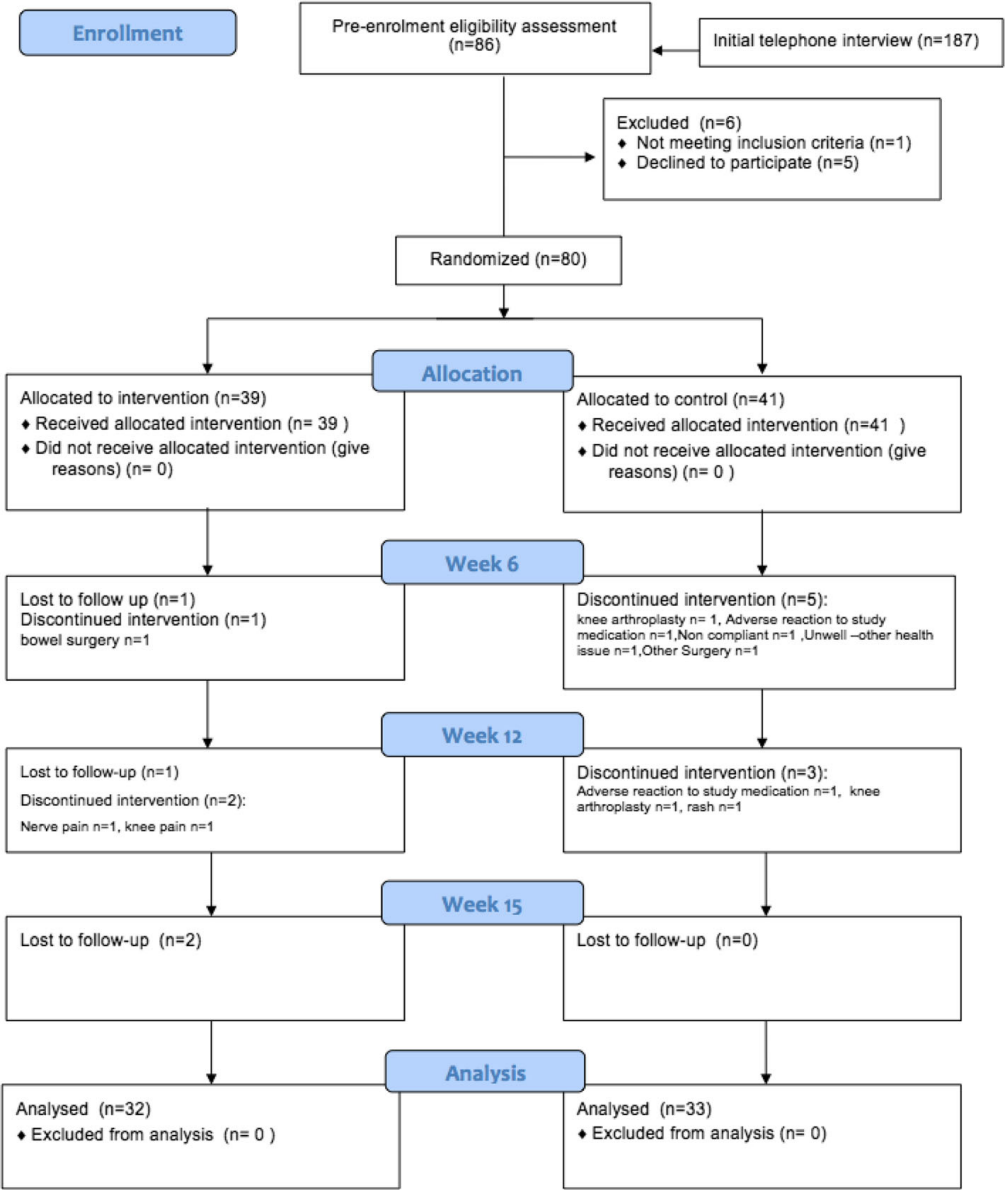
Consort flow diagram of study design and conduct

Initial screening of 187 potential participants was performed by telephone interview. From this group 81 participants were invited to a pre-enrollment assessment visit and 80 were randomized to participate in the study. Five eligible participants declined to participate, giving a 94% (80/85) participation rate. Participants were identified from a variety of sources including advertisements in local newspapers, (*n* = 26), orthopaedic databases (*n* = 15), previous study database (*n* = 14), word of mouth (*n* = 8), Rheumatology outpatients (*n* = 6), Arthritis New Zealand website (*n* = 1), hospital outpatients (*n* = 1), hospital flyer (*n* = 1), with 8 participants not providing this information. Baseline demographics are shown in Table 1. In Fig. 1 the CONSORT diagram shows the path of patients screened and recruited into the randomized controlled trial.

**Table 1:** Participant demographics

	Overall (*n* = 80)	Intervention (*n* = 39)	Control (*n* = 41)
Sex-male^a^	36 (45)	17 (44)	19 (46)
Age^b^	66.4 (10.0)	66.5 (10.8)	66.3 (9.3)
Ethnicity (total response^d^)^a^			
European	77 (96)	38 (97)	39 (95)
Māori	4 (5)	1 (3)	3 (7)
Pacific Peoples	1 (1)	0	1 (2)
BMI^b^	30.1 (5.4)	29.4 (5.1)	30.7 (5.6)
Pre-enrolment pain scale^b^	5.3 (2.0)	5.4 (2.0)	5.2 (2.0)
Pre-enrolment HAQ (Anglicized)^c^	0.75 (0.75)	0.75 (0.50)	0.75 (1.0)
Kellgren-Lawrence x-ray grade^a^			
1	7 (9)	3 (7)	4 (10)
2	15 (19)	6 (15)	9 (22)
3	18 (23)	12 (31)	6 (15)
4	40 (50)	18 (46)	22 (54)
Pre-enrolment Global assessment^b^			
physician	5.7 (1.4)	5.7 (1.3)	5.7 (1.5)
patient	5.2 (1.8)	5.3 (1.8)	5.2 (1.8)
*missing*	*1*	*0*	*1*

^a^n (%) ^b^mean (SD) ^c^median (IQR) ^d^Percentages could add to more than 100 as participants could identify with more than one ethnicity

At screening, 34 participants (42.5%) reported taking CAM medicines (44 never took CAM medicines and 2 did not specify). The most common CAM medicines reported were: glucosamine (*n* = 19), chondroitin (*n* = 7), fish oil (*n* = 20), flaxseed oil (*n* = 3), vitamins/minerals (*n* = 5). All participants discontinued these supplements as required by the protocol, 3 weeks before their baseline assessment.

The baseline demographics of the participants are presented in Table 1.

No evidence was found to suggest that participants could deduce which arm of the study they were in (*p* = 0.688), with the majority (48/66, 73%) of participants believing they were in the placebo arm of the study, (half were in the intervention arm and the other half in the control arm).

### Adherence to study medication

Adherence was generally high (median 79%) and did not appear to differ by group (*p* = 0.205). The per-protocol analysis included 47participants who achieved at least 80% adherence in their medication diary (71%) and also did not appear to differ by group (*p* = 0.274).

### Self-assessment questionnaires

There was no significant difference between groups for; physician global assessment, patient global assessment, pain VAS, OA QoL, HAQ, systolic, WOMAC-total, WOMAC-A, or WOMAC-C (Tables 2 and 3 show results for the total sample).

**Table 2:** Physical and laboratory outcomes (mean (SD) unless otherwise indicated)

	n	Biolex-GLM				Control				*p*-value
Week		0		12		0		12		
Walking (sec)	62	42.7	(12.3)	39.5	(8.9)	41.1	(8.3)	39.2	(6.6)	0.812
Dynamic balance (sec)	50	31.9	(10.7)	28.5	(8.3)	27.8	(9.3)	24.1	(5.9)	0.165
Dynamic balance errors	50	1.6	(2.1)	2.0	(2.2)	1.7	(1.7)	1.2	(1.7)	0.279
Stair climb (sec)	60	13.2	(6.2)	10.2	(4.2)	11.6	(4.7)	10.9	(4.5)	0.207
Sit-to-stand	59	9.3	(3.3)	9.7	(2.8)	9.1	(2.9)	10.6	(2.6)	0.406
Systolic blood pressure	65	133.0	(20.5)	128.0	(25.1)	139.0	(19.2)	133.0	(21.2)	0.858
Diastolic blood pressure	65	77.5	(13.7)	74.9	(14.5)	78.0	(9.1)	78.0	(12.2)	0.140
Cholesterol	41	5.2	(1.1)	5.2	(1.0)	4.8	(0.9)	4.9	(0.8)	0.382
HDL	41	1.5	(0.5)	1.6	(0.4)	1.5	(0.4)	1.6	(0.5)	0.771
Cholesterol:HDL	41	3.6	(0.8)	3.3	(0.8)	3.3	(1.2)	3.3	(1.0)	0.154
LDL	41	3.1	(0.9)	3.0	(0.8)	2.7	(0.8)	2.7	(0.7)	0.291
CRP^a^	64	1.9	(2.1)	1.7	(2.2)	1.9	(2.0)	2.0	(2.1)	0.297
Trigylcerides^a^	41	1.3	(1.5)	1.2	(1.5)	1.3	(1.7)	1.2	(1.6)	0.994

^a^Geometric mean and geometric standard deviation

**Table 3:** Questionnaire (mean (SD) unless otherwise indicated)

	n	Biolex								Control								*p*-value
Week		0		6		12		15		0		6		12		15		
Pain VAS	75	5.3	(2.1)	4.6	(2.4)	5.0	(2.8)	5.2	(2.4)	4.5	(2.1)	4.9	(2.0)	4.6	(2.3)	4.3	(2.1)	0.110
WOMAC-A	75	7.7	(3.4)	7.2	(4.2)	6.6	(4.5)	6.5	(4.2)	8.0	(3.2)	7.6	(3.5)	7.4	(4.2)	7.3	(3.5)	0.955
WOMAC-B	75	3.5	(1.6)	3.7	(1.6)	3.5	(2.0)	3.0	(1.6)	3.9	(1.4)	3.6	(1.9)	3.3	(1.6)	3.7	(1.4)	0.046
WOMAC-C	75	26.0	(14.0)	27.0	(14.0)	24.0	(15.0)	23.0	(13.0)	28.0	(10.0)	26.0	(14.0)	24.0	(12.0)	25.0	(13.0)	0.798
WOMAC-Total	75	37.0	(17.0)	38.0	(19.0)	34.0	(21.0)	32.0	(18.0)	40.0	(14.0)	38.0	(18.0)	34.0	(17.0)	36.0	(17.0)	0.884
OA QoL	75	7.4	(5.9)	7.1	(5.5)	6.6	(6.0)	6.2	(5.4)	7.5	(5.6)	7.0	(6.0)	6.4	(6.0)	6.8	(6.3)	0.805
Physician global assessment	67	5.7	(1.3)			5.6	(1.9)	5.7	(1.9)	5.7	(1.5)			5.6	(1.9)	5.3	(1.5)	0.312
Patient global assessment	67	5.3	(1.8)			4.9	(2.3)	5.4	(1.9)	5.2	(1.8)			4.7	(2.0)	4.8	(1.8)	0.101
HAQ^a^	68	0.9	(0.6)			0.8	(0.6)			0.9	(0.6)			0.8	(0.6)			0.864

^a^Median (IQR)

**Fig. 2:**
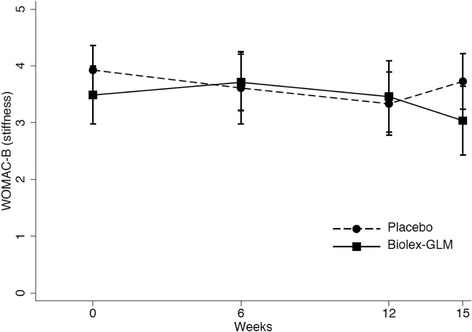
WOMAC-B (stiffness) in Placebo and Biolex- GLM treated groups over course of trial

Over the course of the study, however, a significant difference was noted in WOMAC-B (stiffness) between groups (overall *p* = 0.046 -Table 3 and Fig. 2). This change was evident in the post-intervention period (week 12 to week 15- *p* = 0.016) with the treatment group improving by 0.72 (95% CI 0.14–1.31) compared to control. The magnitude of this change was small (mean 0.5 on a ten unit scale) and is unlikely to indicate a clinically important change in stiffness. Analysis of individual variation suggested a sub group of responders may have accounted for this improvement.

### Changes in analgesic and NSAID use over the course of the study

**Fig. 3:**
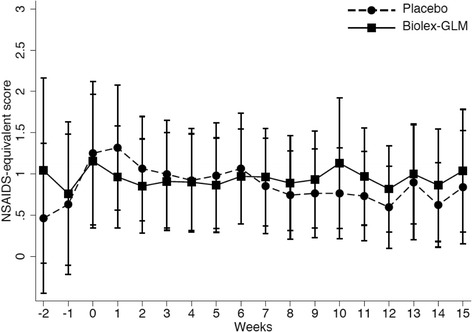
Medication use over course of trial: NSAID equivalents (ASAS formula)

**Fig. 4:**
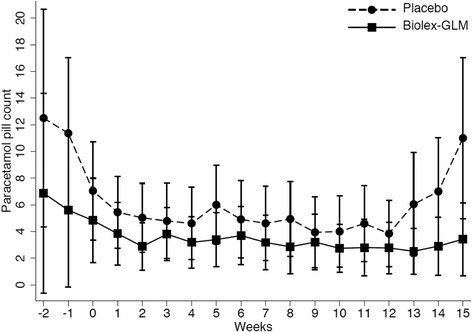
Medication use: Paracetamol tablets taken over course of trial

Analysis of analgesic and NSAID use over the course of the study is shown in Figs. 3 and 4. Use of NSAIDs was very low at baseline. There was a tendency for a difference in slopes for NSAID-equivalent scores from a random coefficients model with the slope (per week) from week 0 to week 12 for the control group being 0.04 lower than for the intervention group (95% CI −0.00–0.09, *p* = 0.067), but no evidence of a change in slopes following week 12 (*p* = 0.353). There was no evidence of a difference in paracetamol use during the study (weeks 0 to 12, *p* = 0.589), but in the post intervention phase after 12 weeks (*p* = 0.001) the placebo group returned to baseline levels of paracetamol intake, whilst the GLM group continued to take fewer paracetamol, this resulted a significant difference between the groups at conclusion of the study (*p* = 0.045).

### Responder criteria

**Fig. 5:**
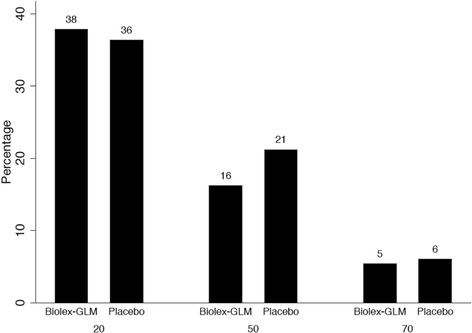
WOMAC total score used to calculate WOMAC-Response criteria: WOMAC-20, 50, 70 outcomes for 70 participants with baseline and week 12 data

Responder criteria were assessed using WOMAC-total as shown in Fig. 5; 37% (26/70) of participants experienced a 20% or greater improvement (14/37 or 38% of Biolex®-GLM; 12/33 or 36% of Placebo; no evidence of a difference [*p* = 0.899 from logistic regression]).

Using WOMAC-A (pain), 41% (29/70) of participants experienced a 20% or greater improvement (Biolex®-GLM: 49%, 18/37; control: 33%, 11/33; no evidence of a difference *p* = 0.196 from logistic regression).

Using WOMAC-A (pain) combined with either WOMAC-B (stiffness) or WOMAC-C (disability, 33% (23/70) participants experienced a 20% or greater improvement in symptoms (Biolex®-GLM: 35%, 13/37; control: 30%, 10/33; no evidence of a difference *p* = 0.668 from logistic regression).

Repeating the analysis for 50 and 70% improvements showed no statistically significant differences between placebo and active treatment for these responders.

### Laboratory analyses-CRP and fasting lipids

CRP and triglycerides were both log-transformed due to positively skewed residuals. There was no evidence of a change between groups among the total sample for total cholesterol, LDL, HDL, total cholesterol:HDL, triglycerides, CRP (Table 2).

### Physical performance testing and blood pressure

There was no evidence of a difference between groups among the total sample for walking time, sit to stand time, stair climb, dynamic balance, dynamic balance errors or systolic and diastolic blood pressure (Table 2).

### Adverse events

Four recorded adverse events were recorded which led to discontinuation of the study medication. Three of these occurred in participants in the placebo group. One event in the Biolex®-GLM treated group required hospital admission. The participant developed abdominal pain requiring hospital admission 8 days after commencing the Biolex®-GLM. The participant had a long standing stoma. It was judged that the event was unlikely to be related to the study medication. The participant was discharged from hospital after 24 h, but was withdrawn from study. In the placebo group the three adverse events were as follows: 1) Participant developed generalized aching at week 7 of study, with flu like symptoms and withdrew from study. 2) Participant developed bilateral pulmonary emboli 24 days after commencing placebo. Participant had poor mobility due to OA. Discharged from hospital after 5 days on warfarin and withdrew from study. 3) Participant developed extensive generalized itchy maculo-papular rash 62 days after commencing placebo. Rash persisted for 3 weeks. Participant was treated with antihistamine and withdrew from study. Rash settled after 10 days.

## Discussion

This randomized double-blind placebo controlled trial, investigated the effects of a novel GLM extract on subjective pain in subjects with moderate to severe OA of the hip and knee, and is the first to use OARSI/OMERACT recommended outcome measures including pain and global assessment together with responder criteria. Furthermore, a minimum level of pain was a specified criteria for enrollment in the study and obviated a floor effect in response (OA subjects with mild pain, < 30 mm VAS, were excluded). The study was designed to be sufficiently powered and of sufficient duration to detect a clinically meaningful difference in the primary outcome measures. While the sample size calculated was not achieved, the effect on the detectable difference in VAS pain scores would have been increased only slightly from 13 mm to 14 mm. The actual power of the study is reflected in the widths of the presented confidence intervals.

The current study showed no benefit for Biolex®-GLM over placebo for either of the primary outcomes; WOMAC- pain score, VAS pain score and there was no evidence of a response at the 20% improvement level for WOMAC pain or WOMAC –total score. Secondary outcomes including patient global assessment, OAQol, HAQ disability score and a range of physical performance tests also showed no significant benefit for GLM over placebo.

Changes over the study period in 2 outcome measures were of interest. Firstly patients in the Biolex®-GLM treated arm of the study reduced their intake of paracetamol in contrast to the placebo group and this persisted after the intervention ceased at week 12. Although the reduction in analgesic use was not significant over the whole study period (*p* = 0.589) there was a significant difference after discontinuation of the active treatment (*p* = 0.001). This finding raises the possibility of persisting benefit in the Biolex®-GLM treated group.

Secondly, there was a small reduction in stiffness measured by WOMAC-B in the Biolex®-GLM treated group between 12 and 15 weeks post-intervention. Although statistically significant (*p* < 0.05), the level of this reduction was unlikely to be clinically meaningful.

To our knowledge, this is the first study to use NSAID equivalent dose calculations to assess the effect on NSAID use for an intervention in OA. The Biolex®-GLM extract did not reduce the use of NSAIDs compared with placebo over the study period. The Biolex®-GLM product did not alter lipid levels or CRP over the course of the study.

The GLM extract appeared safe with no attributable serious or significant adverse events over the15 week study period.

Previous studies have investigated a variety of GLM products, both lipid extracts and powdered products. These products differ in their in vitro efficacy. To date there have been three randomized controlled trials (RCTs) of GLM products in OA [24–27]: all claim benefit for at least one outcome measure selected. All used a VAS pain scale as an outcome. Gibson et al., [25] and Audeval et al. [26] showed no significant improvement in pain, but Lau et al. [27] did show a significant improvements in pain, although the mean improvement (9.0 mm) is not recognized as clinically meaningful and no minimum pain level was set as a criterion for entry to the study.

In addition, there have been 3 systematic reviews of GLM products for the treatment of OA [7, 8, 28], where these 3 RCT’s have been assessed critically. The use of different extraction processes and variable durations have resulted in heterogeneous studies of variable quality. Small numbers of participants (*n* = 27 to *n* = 80) and a lack of a priori power calculations are also evident in the literature. There was a high risk of bias for these studies and a meta-analysis of pooled data or results was not feasible. A more recent open label pilot study [24] used the WOMAC scale as an outcome measure. In total, 21 patients completed 8 weeks on the GLM preparation. Significant improvements from baseline were noted in the WOMAC total and all 3 subdivisions of the scale. Thus to date, there is limited evidence that GLM extracts reduce pain or improve functional outcomes in OA.

The current study has limitations. Firstly, half the participants had severe OA (Kellgren-Lawrence grade 4 radiographic changes). This may have affected the ability to detect improvements in self-reported pain, and more specifically physical function outcomes. Secondly, the recording of NSAID use as an outcome measure was hindered by the surprisingly low use of NSAIDs amongst the study participants, despite high baseline pain scores. Thirdly, as with other studies in OA, the control group demonstrated a prominent placebo response raising the threshold to detect significant differences in pain levels. Fourthly, the sample size calculated was not achieved but this had minimal effect on the detectable difference for VAS pain scores.

Future studies may wish to address some methodological limitations which became apparent on conclusion of this study. Firstly, it was decided not to undertake an initial pilot study given the previous literature of mostly small studies of GLM extracts. Dosage was based on previous studies of GLM extract containing a different profile of lipids compared to Biolex®-GLM. We therefore cannot exclude the possibility that a higher dose of the Biolex®-GLM extract may have had a greater effect. Secondly, the final follow up assessment was 3 weeks after discontinuation of the study medications. It would have been interesting to see if apparent sustained improvements in some outcomes were maintained beyond this period and for how long. Thirdly, a longer duration of treatment may have been beneficial, and the trends towards the end of the study suggest that this is worth exploring in future studies.

## Conclusions

In conclusion, in this double blind randomized placebo controlled study of a novel Biolex®-GLM extract administered over 12 weeks the GLM extract appeared safe with no apparent adverse effects. There was no significant improvement in the primary outcome of pain in moderate to severe OA, but there was a significant difference in the use of paracetamol in those taking the Biolex®-GLM extract in the 3 week post treatment period, due to the placebo group increasing their paracetamol use to baseline levels, but the Biolex®-GLM maintaining a lower intake. This period was also associated with a significant reduction in stiffness.

Further studies with higher doses of Biolex®-GLM extract for a longer duration are worthy of consideration, particularly to investigate the potential to reduce reliance on paracetamol for analgesia.

## Abbreviations

CAM: Complimentary and alternative medicine
CRP: C-reactive protein
GLM: Green lipped mussel
HAQ: Health assessment questionnaire disability index
NSAID: Non-steroidal anti-inflammatory drug
OA: Osteoarthritis
OAQoL: Osteoarthritis quality of life index
OARSI: Osteoarthritis Research Society International
OMERACT: Outcome measures in rheumatology clinical trials
STS: Sit to stand test
WOMAC: Western Ontario and McMasters OA Index
